# Neuropsychological Consequences for Survivors of Childhood Brain Tumor in Malaysia

**DOI:** 10.3389/fpsyg.2018.00703

**Published:** 2018-05-29

**Authors:** Hamidah Alias, Sie Chong D. Lau, Ilse Schuitema, Leo M. J. de Sonneville

**Affiliations:** ^1^Department of Pediatrics, UKM Medical Center, Faculty of Medicine, The National University of Malaysia, Kuala Lumpur, Malaysia; ^2^Department of Clinical Child and Adolescent Studies, Faculty of Social Sciences, Leiden University, Leiden, Netherlands

**Keywords:** Amsterdam Neuropsychological Task (ANT), children, brain tumor, neuropsychological sequelae, survivors

## Abstract

**Objective:** This study aimed to evaluate neuropsychological consequences in survivors of childhood brain tumor.

**Method:** A case-control study was conducted over a period of 4 months in a tertiary referral center in Kuala Lumpur, Malaysia. Fourteen survivors of childhood brain tumor aged 7–18 years, who were off-treatment for at least 1 year and were in remission, and 31 unrelated healthy controls were recruited. The median age at diagnosis was 8.20 years (range: 0.92–12.96 years). The diagnoses of brain tumors were medulloblastoma, germ cell tumor, pineocytoma, pilocystic astrocytoma, suprasellar germinoma, and ependymoma. Eleven survivors received central nervous system irradiation. Seven tasks were selected from the Amsterdam Neuropsychological Tasks program to evaluate alertness (processing speed), and major aspects of executive functioning, such as working memory capacity, inhibition, cognitive flexibility, and sustained attention. Speed, stability and accuracy of responses were the main outcome measures.

**Results:** Survivors of childhood brain tumor showed statistically significant poorer performance on all tasks compared to healthy controls. Both processing speed and accuracy were impaired in the survivors, in particular under more complex task conditions. The survivors demonstrated deficits in alertness, sustained attention, working memory capacity, executive visuomotor control, and cognitive flexibility. Longer duration off treatment appeared to be correlated with poorer alertness, memory capacity, and inhibition.

**Conclusion:** Survivors of childhood brain tumor in our center showed impaired neuropsychological functioning. Development of less toxic treatment protocols is important to prevent late effects of cognitive deficits in survivors of childhood brain tumor.

## Introduction

Primary brain tumors belong to the second most common type of cancer in children, representing about 20% of all pediatric cancers (Ellison et al., 2009; Kaatsch, 2010). Every year, approximately 30,000–40,000 children are diagnosed with brain tumors worldwide (Bleyer, 1999). MB is the most common pediatric brain tumor, representing approximately 30% of all childhood brain tumors and is usually found in the PF (Saletta et al., 2014). Improved surgical treatment and the use of CI and/or chemotherapy have significantly increased the survival rate of these patients. The 5-year overall survival rate ranges from 70 to 80% for standard risk MB (Saletta et al., 2014), although in countries with limited resources lower rates are reported (Rajagopal et al., 2016).

Unfortunately, many of the survivors experience disease-related morbidity and treatment-related neurotoxicity, especially from CI, including neuropsychological dysfunction (Antonini et al., 2017; Fay-McClymont et al., 2017; Raghubar et al., 2017). Deficient development or loss of the normal appearing white matter from treatment, especially CI, and decreased cerebral perfusion were reported to cause progressive cognitive impairment, affecting processing speed, attention and working memory post treatment (Mulhern et al., 2001, 2004a; Fouladi et al., 2004; Mabbott et al., 2008; De Smet et al., 2009; Rueckriegel et al., 2010; Scantlebury et al., 2016). Poor executive function and speed of processing were identified as the ‘core deficit’ by neuropsychological investigation of survivors with childhood brain tumor (Butler and Mulhern, 2005). Deficits in attentional functioning were evident approximately 4–5 years post CI and were associated with reduced volumes of normal-appearing white matter (Dennis et al., 1998; Reddick et al., 2003; Mulhern et al., 2004b). Marked executive dysfunctions have also been reported previously in survivors of PF tumor treated with surgical resection only (Levisohn et al., 2000), or combined with postsurgical adjuvant treatment (Koustenis et al., 2016).

In summary of the literature, risk factors for poorer neuropsychological functioning included RT dose, longer time since treatment, high grade tumor histology, brain volume that received treatment (Mulhern et al., 2004b, 2005; Gerber et al., 2008; Ellenberg et al., 2009; Armstrong et al., 2010; Robinson et al., 2013), and younger age at diagnosis, the latter being the most prominent risk factor (Mulhern et al., 2005), but also the interplay with chemotherapy should also be taken into account (Mulhern et al., 2004a; Bull et al., 2007).

In view of the consequences of treatment-related neurotoxicity, which seems to invariably compromise the integrity of neural functional networks, the present study aimed to identify a profile of executive dysfunctions in children and adolescents that suffered from low- or high-grade cerebellar tumors. Multiple function tests, predominantly used in previous studies, provide outcomes that are the result of multiple functions necessary to execute a certain test. As a result, the outcomes are usually multi interpretable. The ANT program uses task paradigms in which task load (e.g., memory load) is varied. By measuring the effect of this manipulation, differences between comparison groups can be interpreted in terms of the manipulated process by studying the group × task load interactions. In this way the ANT allows to precisely evaluate speed and accuracy of specific aspects of executive function and attention (de Sonneville, 1999). To the best of our knowledge, this approach has been used only once in pediatric cerebellar tumor survivors, using only three tasks of the ANT program (Koustenis et al., 2016). We extended the assessment to seven tasks, enabling to identify a more detailed profile of neuropsychological (dys)functioning, including alertness, working memory, sustained attention, inhibition, cognitive flexibility, and executive visuomotor control in our survivors with childhood brain tumor. From the literature, we hypothesize that the survivors will perform poorly on all evaluated aspects of neuropsychological functioning. We also explored disease-related or treatment-related factors that could cause poor neuropsychological performance in our survivors.

## Materials and Methods

### Subjects

This was a single center, case-control study conducted in Pediatric Hematology & Oncology Unit at Department of Pediatrics, Universiti Kebangsaan Malaysia Medical Center (UKMMC) over a 4-month period from 13th March 2015 until 31st July 2015. All brain tumor survivors aged 7–20 years old who had completed treatment for more than 1 year and remained in remission were eligible for the study. Patients with pre-existing neurological deficit not related to brain tumor disease or treatment or patient with severe hemiparesis, paraplegia or total paralysis which rendered them incapable of completing the tasks involved in the study were excluded. A control group of healthy school children not related to the survivors and randomly selected, and matched for gender and age were recruited from schools in and outside Kuala Lumpur.

Ethics approval was obtained from the Research Ethics Committee Universiti Kebangsaan Malaysia prior to the study (UKM 1.5.3.5/244/FF-2015-066). A written informed consent from the parents was obtained prior to recruitment of subjects.

### Measures

#### Neuropsychological Tasks

Seven tasks were selected from the ANT program and administered in approximately 1 hour, in a fixed order as described below. The ANT is a computerized neuropsychological test battery to enable systematic evaluation of attention and information processing, i.e., the basic processes required for the execution of complex cognitive processes. Numerous studies in various clinical domains have demonstrated a satisfactory sensitivity and validity of the ANT (Swaab-Barneveld et al., 2000; De Sonneville et al., 2002; Huijbregts S. et al., 2002; Huijbregts S.C. et al., 2002; Huijbregts et al., 2010; Barneveld et al., 2013). It had also been used to examine the neuropsychological aspects of childhood leukemia following treatment with CI (Schuitema et al., 2013, 2015) and chemotherapy (Buizer et al., 2005a,b). In the current study each participant was allowed to perform two practice sessions for each task before the actual test was administered. This was to ensure that they fully understood what they were asked to do. In general, during task performance they were instructed to respond as fast and as accurately as possible. All investigators involved in the study were extensively trained to administer the ANT tasks before the study began.

##### Baseline speed (BS)

This simple reaction time task measures alertness, and involves minimal cognitive effort (Konrad et al., 2004). Subjects were required to press a mouse-key with the index finger as quickly as possible when a fixation cross in the center of the computer screen changed into a white square. The task consisted of two parts with 32 trials for the preferred hand and non-preferred hand. The mean speed and within-subject SD of reaction times (fluctuation in speed) were calculated over both the preferred and the non-preferred hand responses.

##### Memory search letters (MSL)

Memory search letters used to measure working memory capacity and distraction (De Sonneville et al., 2002). This letter detection task consists of three parts increasing the memory load from one item in part 1 (*k*), to two items in part 2 (*k*+*r*), and three items (*k*+*r*+*s*) in part 3. The display set of four letters that contains the complete target set requires a ‘yes’-response, incomplete target sets requires a ‘no’-response. Target letters in non-target trials act as distractors. Memory search rate is operationalized as the contrast in speed/accuracy of responses to target signals in part 1 (low load) and part 3 (high load). Distraction is operationalized as the contrast in speed/accuracy of responses to non-target signals in part 3 between signals with 0 distractors (low distraction) and two distractors (high distraction). Reaction time to target signals is predicted to increase linearly with memory load, reflecting the prolongation of the memory search stage, with the slope of reaction time denoting the rate of memory search. In part 3, the presence of an insufficient number of target letters in non-target signal affects response time in that reaction time will increase with the number of these ‘distracters’ (0, 1, or 2). Each part consists of 50% target and 50% non-target signals with 40, 72, and 96 trials in parts 1–3, respectively, with non-target trials evenly divided across distracter type.

##### Sustained attention dots (SAD)

This task measures the ability to maintain performance at a certain level during a longer period of time. During this task 600 random patterns of three, four or five dots are successively presented in 50 series of 12 trials. Subjects are required to respond to the 4-dots pattern (target) by pressing the mouse button with their preferred hand (‘yes’-response) and to the 3- or 5-dots patterns (non-targets) by pressing the mouse with their non-preferred hand (‘no’-response). The ratio targets/non-targets is 1/2 which invokes a response bias for the ‘no-response.’ Failure to inhibit this bias is expected to result in the production of relatively more misses than false alarms. Main outcome measures are the 50 series completion times, and the number and type of errors per series. To obtain measures of sustained attention, i.e., the change (deterioration) of performance level with TOT, the 50 series were divided into five blocks of 10 series each, and mean tempo, within-subject SD of tempo (fluctuation in tempo), and percentage of errors were calculated per block. During performance, subjects were informed about errors by a beep signal. Correct responses following an error were separately registered enabling the measurement of the effect of feedback, which is defined as the difference RT_afterfeedback_ – RT_regular_ (post-error slowing).

##### Tracking (TR)

This task measures accuracy and stability of movement along a planned trajectory. The subject is required to trace the mouse cursor in between the inner circle (radius 7.5 cm) and outer circle (radius 8.5 cm) presented on the computer screen. The cursor had to be moved in clockwise direction once with the right hand and once in counter-clockwise direction with the left hand. The trajectory was divided into 60 radially equal segments and the program computed the mean distance between the cursor trajectory and the midline per segment, resulting in 60 deviation scores. Mean deviation of the moving target (accuracy of movement) from the midline and the within-subject SD of the 60 deviation scores (stability of movement) during TOT were taken as main outcome parameters.

##### Pursuit (PU)

This task demands the concurrent planning and execution of movement. It assesses the quality of executive motor control. Subject is required to continuously track a target moving randomly on the screen, by moving the computer mouse as closely as possible. The task time is 60 s. The program calculates the mean distance between the mouse and the target per second task time, resulting in 60 deviation scores. Main outcome parameters are the mean distance to the target and the within-subject SD of the 60 deviation scores.

##### Shifting attentional set – visual (SSV)

This task assesses cognitive flexibility and inhibition. A colored square moves randomly to the right and to the left of a horizontal bar that is permanently present on the computer screen. The task consists of three parts. In part 1 (fixed compatible condition) the child is asked to follow the movement of a green square by pressing the left button upon a left move and the right button upon a right move. In part 2 (fixed incompatible condition), using a red square, the subject is asked to do the opposite, by pressing the left button upon a right move and vice versa, requiring the inhibition of prepotent responses. Inhibition is operationalized as the contrast in performance (speed/accuracy) between part 1 and part 2. In part 3 (random condition), the block changes color randomly requiring the child to follow or ‘mirror’ the movement, depending on the color of the block. Now the subject needs to shift response sets, i.e., readily switch between execution of a prepotent response and inhibition of a prepotent response, a switch requiring cognitive flexibility. Cognitive flexibility is operationalized as the contrast in performance between part 1 and part 3 (compatible trials). It is expected that the cost of inhibition and flexibility results in slower responses and/or more errors.

##### Visuo-spatial sequencing (VSS)

This task evaluates memory of visuospatial temporal patterns. In each trial, several circles are pointed out in an array of nine circles, arranged in a 3 × 3 matrix on the computer screen. The subject has to point out the same circles in the same order by moving the mouse cursor and must press a button when the cursor is positioned at the right location(s). The test consists of 24 trials in which the number of target circles varies from three to seven and in which the spatial sequential patterns increase gradually in complexity. Outcome measures are the number of correctly completed trials, the number of correctly identified circles, and the number of correctly identified circles in the correct order. Sequential working memory is operationalized as the contrast between these two scores.

Examples of stimuli and timing between signals can be found in Koekkoek et al. (2006) for tasks BS, PU and TR, for tasks SAD, MSL, and SSV in De Sonneville et al. (2002), and for task VSS in Schuitema et al. (2015). Test–retest reliability and validity of the ANT are satisfactory and have extensively been described (De Sonneville et al., 2002, Günther et al., 2005; Rowbotham et al., 2009; De Sonneville, 2014). Mean test–retest reliability for the variables varies, depending on task between 0.73 and 0.80 (De Sonneville, 2014).

### Data Analysis

Tests of homogeneity of variance and normality were performed and assumptions were met. Results were evaluated by GLM repeated measures ANOVA (RM-ANOVAs) with group (controls vs. survivors) as between-subjects factor using the SPSS program version 20. Because reaction time (but not accuracy) in ANT tasks is significantly correlated with age (De Sonneville, 2014), age was entered as a covariate in the reaction time analyses. The various task manipulations were used as levels of within-subject (WS) factors and are indicated per task. MSL: *memory load* (parts 1–3), *distraction* within part 3 (0–2 distracters); SAD: *time-on-task* (period 1–5), *bias* (misses vs. false alarms); SSV: *attentional flexibility* [not-required (part 1)], – required (part 3, compatible trials), and *inhibition* [compatible (part 1)], incompatible (part 2); VSS: *recall criterion* (correct order relevant vs. irrelevant). Mean speed and fluctuation in speed in task BS were entered in a multivariate analysis of covariance. Mean tempo, fluctuation in tempo, mean error rate, and post-error slowing in task SAD were entered in a multivariate analysis of (co)variance. Mean deviation and fluctuation in deviation were entered in RM-ANOVAs, respectively, wherein *task type* (TR vs. PU) was entered as a WS factor. In the RM-ANOVAs, the focus will particularly be on the group × task manipulation interactions. Significant interaction effects indicate that the task effect on performance is different for the two groups allowing us to interpret the group differences in terms of the manipulated process/function.

Subjects were excluded from task analysis when their error rate was >50% or in case of extreme mean reaction time values (>3× the IQR). Two patients and one control subject were excluded for task MSL, SSV, and SAD, one patient for task VSS, and there were no exclusions for the other tasks.

Correlational analyses were performed to explore possible associations of age at diagnosis and duration off treatment with task performance. For these analyses *z*-scores of task performance were entered to control for age of the survivors. These *z*-scores are automatically computed by means of non-linear regression functions that describe task performance as a function of age. These regression functions, intrinsically implemented in the ANT program, are based on norm samples consisting of 6.770 (BS), 3.240 (MSL), 2.340 (PU), 3.260 (TR), 3.180 (SAD), 830 (VSS), and 3.440 (SSV) of typically developing subjects and are therefore considered to be reliable estimates of performance level (De Sonneville, 2014).

In order to facilitate a clinical impression of the survivors performance, we assigned the survivors to one of the following three categories: z < 1 (within the normal range or better than the norm), 1 ≤ z ≤ 2 (between 1 and 2 SD deviating from – poorer than – the norm), z > 2 (more than 2 SD deviating from the norm). To this purpose we computed mean *z*-scores for reaction time, errors and fluctuation in reaction time per task. Categorical data were presented in frequency and percentage, and analyzed using chi-square test. Mann–Whitney *U* Test was used for data that was not normally distributed. Significance level was set at *p*-value of <0.05. Effect sizes were calculated using partial eta squared with $\eta_{p}^{2}$ ∼ 0.03 representing a weak effect, $\eta_{p}^{2}$ ∼ 0.06 representing a moderate effect and $\eta_{p}^{2}$ ≥ 0.14 significantly a large effect (Cohen, 1992).

## Results

### Subjects

A total of 28 survivors with childhood brain tumor were identified from the unit’s database. Seven of 28 survivors were excluded as they did not fulfill the inclusion criteria while another seven survivors were lost to follow-up. There were no significant differences between survivors with childhood brain tumor and healthy controls in the demographic variables except for the fathers’ education level (**Table 1**). The median age of the survivors at diagnosis was 8.20 years (IQR 25th 5.76, 75th 10.51) while median duration off treatment was 6.37 years (IQR 25th 2.44, 75th 9.57). Disease and treatment characteristics of survivors with childhood brain tumor are shown in **Table 2**. The ANT tasks were performed by 14 survivors (nine males and five females) and 31 unrelated healthy controls. One of the survivors was only able to complete four out of the seven tasks administered.

**Table 1:** Demographic characteristics of study population.

Characteristics	Childhood brain tumor survivors (*n* = 14)	Healthy controls (*n* = 31)	*p*-value
Median age at study entry,	16.00	15.14	0.177
year (IQR)	(25th 14.03; 75th 17.89)	(25th 12.49; 75th 17.30)	
Gender, *n* (%)			0.820
Male	9 (64.3)	21 (67.7)	
Female	5 (35.7)	10 (32.3)	
Education, *n* (%)			
Primary	3 (21.4)	5 (16.1)	0.279
Secondary	10 (71.4)	26 (83.9)	
Special	1 (7.1)	0 (0)	
Father’s median age,	46.5	49.0	0.463
year (IQR)	(25th 44.5; 75th 54.5)	(25th 41.0; 75th 50.0)	
Mother’s median age,	45.50	47.0	0.344
year (IQR)	(25th 42.7; 75th 52.7)	(25th 40.0; 75th 48.0)	
Father’s education level, *n* (%)			
Primary	0 (0)	2 (6.5)	0.014
Secondary	10 (71.4)	8 (25.8)	
Tertiary	4 (28.6)	21 (67.7)	
Mother’s education level, *n* (%)			
Primary	1 (7.1)	1 (3.2)	0.662
Secondary	8 (57.1)	15 (48.4))	
Tertiary	5 (35.7)	15 (48.4)	
Family monthly income, *n* (%)			
<MYR3000	2 (14.3)	5 (16.1)	0.955
MYR3000–MYR7000	7 (50.0)	14 (45.2)	
>MYR7000	5 (35.7)	12 (38.7)	
Number of siblings, *n* (%)			
<2	1 (7.1)	7 (22.6)	0.309
2–5	8 (57.1)	18 (58.1)	
>5	5 (35.7)	6 (19.4)	
Siblings with chronic illness or disability, *n* (%)			
No	13 (92.9)	30 (96.8)	0.262
Yes	1 (7.1)	1 (3.2)	

*IQR, interquartile range; n, number; MYR, ringgit Malaysia.*

**Table 2:** Disease and treatment characteristics of survivors of childhood brain tumor.

No.	Gender	Age at diagnosis, years	Age at study entry, years	Diagnosis	Surgery (no.)	VP shunt	Radiation, Gy	Chemotherapy protocol (cumulative dose of chemotherapy)	Disability
1	Male	5.83	19.04	Medulloblastoma (std risk)	Total resection	Yes	CSI 35Gy/PFB 18Gy	None	V
2	Male	4.89	17.51	Medulloblastoma (high risk)	Subtotal resection	Yes	CSI 35Gy/PFB 18Gy Whole spine boost 7.2Gy	Australia + New Zealand Protocol Methotrexate 48,000 mg/m^2^	–
3	Male	5.52	16.99	Pineal gland GCT (malignant teratoma + embryonal carcinoma + choriocarcinoma)	Near-total resection	Yes	CSI 27Gy/WB boost 27Gy	BEP protocol Cisplatin 400 mg/m^2^	V/H/M
4	Male	10.36	20.97	Medulloblastoma (high risk)	Subtotal resection	No	CSI 36Gy/PFB 18Gy	HIT-SIOP PNET 4 protocol Cisplatin 450 mg/m^2^	V/H
5	Female	0.92	11.45	Pineocytoma	Subtotal resection	No	None	None	–
6	Male	10.83	19.60	Left parietal lobe germinoma	Partial resection	Yes	CSI 30Gy/WB boost 16Gy	SIOP GCT 96 protocol Cisplatin 600 mg/m^2^ Ifosfamide 45,000 mg/m^2^	M
7	Female	7.51	16.14	Pilocytic astrocytoma (relapse)	Total resection	No	None	None	–
8	Female	8.57	16.75	Medulloblastoma (high risk)	Subtotal resection	Yes	CSI 36Gy/PFB 18Gy Spine (C5-T4) boost 14.4Gy	HIT-SIOP PNET 4 protocol Cisplatin 600 mg/m^2^	V/H/M
9	Female	7.78	14.33	Suprasellar germinoma	Subtotal resection	No	CSI 24 Gy/WB boost 18Gy	None	V
10	Male	7.84	13.14	Ependymoma	Subtotal resection	Yes	None	None	V/M
11	Female	11.17	15.79	Medulloblastoma (std risk)	Subtotal resection	No	CSI 23.4Gy/PFB 30.6Gy	HIT-SIOP PNET 4 protocol Cisplatin 560 mg/m^2^	V/H/M
12	Male	10.41	15.46	Medulloblastoma (High Risk)	Subtotal resection	Yes	CSI 35Gy/PFB 19.8Gy	None	–
13	Male	8.75	11.95	Third ventricle immature teratoma	Subtotal resection	No	CSI 34.2Gy/WB boost 16.2Gy	SIOP GCT 96 protocol Cisplatin 400 mg/m^2^ Ifosfamide 30,000 mg/m^2^	–
14	Male	12.96	15.81	Medulloblastoma (std risk)	Total resection	No	CSI 35Gy/PFB 18Gy	HIT-SIOP PNET 4 protocol Cisplatin 490 mg/m^2^	V/H

*GCT, germ cell tumor; std, standard; VP, ventriculoperitoneal; CSI, craniospinal irradiation; PFB, posterior fossa boost; WB, whole brain; V, vision; H, hearing; M, motor.*

Following surgery, three survivors did not require any further treatments; two survivors received RT; nine survivors received a combination of RT and chemotherapy. Two survivors received high dose intravenous ifosfamide with a mean cumulative dose of 37,500 mg/m^2^ and nine survivors received intravenous cisplatin with a mean cumulative dose of 546.6 mg/m^2^. Nine survivors had long-term disabilities post-treatment, namely physical, visual or hearing disabilities. Six of them had more than one disability. The disabilities documented were unsteady gait and mild unilateral hemiparesis, unilateral esotropia, unilateral nystagmus, unilateral or bilateral hemianopia, unilateral conductive hearing loss and unilateral low frequency hearing loss. However, the degree of disability did not interfere with their ability to perform or complete the ANT tests.

#### Baseline Speed

The survivors were significantly slower with mean reaction speed of 375 ± 88 ms compared to the controls, 285 ± 25 ms, [*F*(1,40) = 25.758, *p* < 0.0001, $\eta_{p}^{2}$ = 0.392]. The survivors also showed more fluctuation in reaction speed with a mean SD of 124 ± 55 ms compared to the controls, 66 ± 37 ms, [*F*(1,40) = 15.809, *p* = 0.0001, $\eta_{p}^{2}$ = 0.283].

#### Memory Search Letters

**FIGURE 1:**
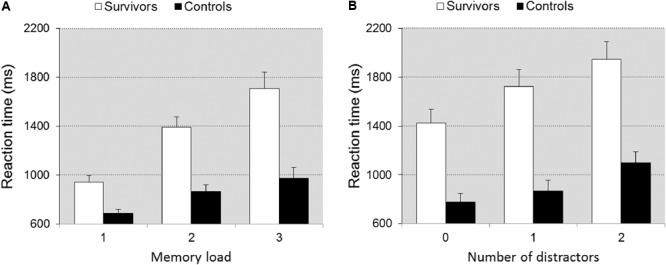
Reaction time ± SEM as a function of memory load **(A)** and distraction **(B)** in task MSL. Illustrates the significant group × memory load/distraction interaction. The impact of the increase in memory load/distraction is larger in the survivors.

The survivors were significantly slower than controls [*F*(1,39) = 23.181, *p* < 0.0001, $\eta_{p}^{2}$ = 0.373] and group interacted with memory load [*F*(2,78) = 16.307, *p* < 0.0001, $\eta_{p}^{2}$ = 0.295], reflecting that differences in speed between survivors and controls increased with memory load (**Figure 1A**). The data analyses of task part 3 shows that group differed in speed [*F*(1,39) = 25.008, *p* < 0.0001, $\eta_{p}^{2}$ = 0.391] with survivors being slower. Group interacted with distraction [*F*(2,78) = 8.983, *p* < 0.001, $\eta_{p}^{2}$ = 0.187] indicating that differences between survivors and controls became larger with increasing distraction (**Figure 1B**). Both manipulations imposed high demands on the working memory system. The analyses on error rates did not reveal any significant differences between groups (0.067 < *p* < 0.502).

#### Pursuit and Tracking

**FIGURE 2:**
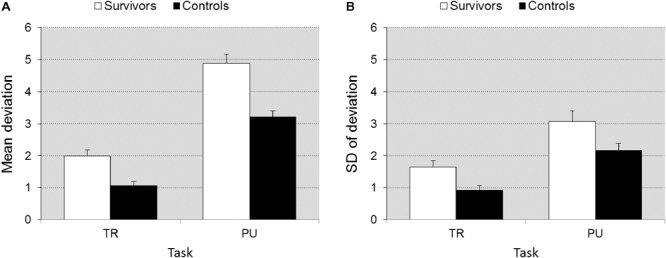
Accuracy ± SEM **(A)** and fluctuation in accuracy ± SEM **(B)** as a function of task (TR vs. PU). Illustrates the significant group × task interaction. The differences between groups is larger on task PU (higher executive function demands) than on TR (lower EF demands).

Groups differed in accuracy [*F*(1,39) = 33.00, *p* < 0.0001, $\eta_{p}^{2}$ = 0.458]. The differences between groups were larger on PU than TR [*F*(1,39) = 7.636, *p =* 0.009, $\eta_{p}^{2}$ = 0.164], reflecting that when executive function demands were higher, differences between groups became larger (**Figure 2A**). The survivors also showed more fluctuation in accuracy than controls [*F*(1,39) = 25.85, *p* < 0.0001] and again differences were larger on PU vs. TR [*F*(1,39) = 6.574, *p* < 0.014, $\eta_{p}^{2}$ = 0.144] (**Figure 2B**).

#### Sustained Attention Dots

**FIGURE 3:**
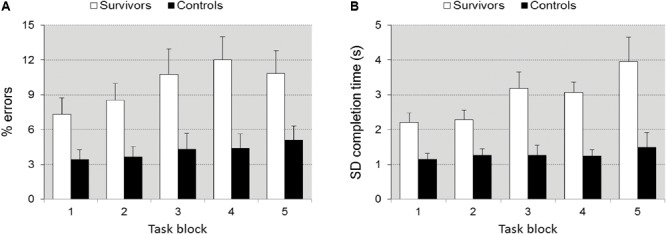
Accuracy ± SEM **(A)** and fluctuation in tempo ± SEM **(B)** as a function of time-on-task in task SAD. Illustrates the significant group × TOT interaction. The increase in errors and fluctuation in tempo with TOT is larger in the survivors.

Tempo, fluctuation in tempo, and error rate differed significantly between groups [*F*(1,38) = 35.607, *p* < 0.0001, $\eta_{p}^{2}$ = 0.484], [*F*(1,38) = 19.199, *p* < 0.0001, $\eta_{p}^{2}$ = 0.336], and [*F*(1,38) = 4.905, *p* = 0.033, $\eta_{p}^{2}$ = 0.114], respectively. The survivors were slower (15.29 ± 4.74 s vs. 9.59 ± 1.96 s), had a higher fluctuation in tempo (2.57 ± 1.18 s vs. 1.43 ± 0.71 s), and made more errors than controls (8.56 ± 7.24% vs. 5.41 ± 2.90%). The group by TOT interaction approached significance for error rate [*F*(4,152) = 2.081, *p* = 0.086, $\eta_{p}^{2}$ = 0.052], including a *post hoc* simple contrast of task block 4 vs. 1 (*p* = 0.016, $\eta_{p}^{2}$ = 0.052), and for fluctuation in tempo [*F*(4,152) = 2.29, *p* = 0.062, $\eta_{p}^{2}$ = 0.057], including a *post hoc* simple contrast of task block 4 vs. 1 (*p* = 0.015, $\eta_{p}^{2}$ = 0.057) but not for tempo (*p* = 0.242), suggesting a larger deterioration of fluctuation in accuracy (**Figure 3A**) and tempo (**Figure 3B**) in the survivors (**Figure 3**). Differences between groups in response bias were not significant (*p* = 0.291). Post-error slowing (422 ms ± 267 for controls, 587 ± 321 for survivors) did not differ significantly (*p* = 0.291).

#### Visuo-Spatial Sequencing

**FIGURE 4:**
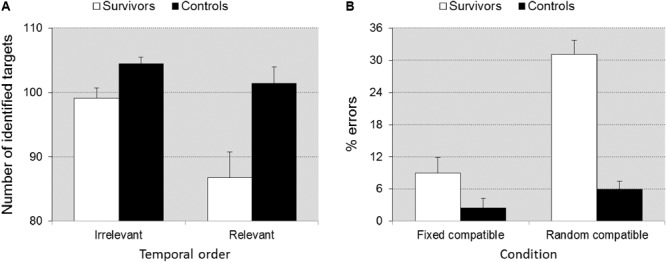
Memory score ± SEM in task VSS as a function of scoring criterion **(A)**, accuracy ± SEM in task SSV as a function of task condition **(B)**. Illustrates the significant group × scoring criterion interaction **(A)** and the group × task condition interaction **(B)**. When temporal order of the recall is relevant (higher working memory load), the difference between groups is larger. Under the random compatible task condition (cognitive flexibility required) the differences between groups is larger than under the fixed compatible condition (flexibility not required).

Group differences were significant [*F*(1,39) = 14.26, *p* = 0.001, $\eta_{p}^{2}$ = 0.268]. The number of identified targets was higher for the control group compared to the survivors. Group interacted significantly with Recall criterion (correct temporal order relevant vs. irrelevant) [*F*(1,39) = 7.989, *p* = 0.007, $\eta_{p}^{2}$ = 0.170], indicating that differences in accuracy increased when working memory demands were higher (reproduction of visuospatial location *and* temporal order) (**Figure 4A**).

#### Shifting Attentional Set – Visual

##### Inhibition

Survivors were overall slower than controls [*F*(1,41) = 13.927, *p =* 0.001, $\eta_{p}^{2}$ = 0.254] (727 ± 319 ms vs. 501 ± 115 ms), but the group × inhibition interaction was only trend significant [*F*(1,41) = 3.563, *p =* 0.066, $\eta_{p}^{2}$ = 0.080], suggesting that differences in speed between groups increased only slightly when inhibition was required. Survivors made more errors than controls [*F*(1,41) = 14.033, *p* = 0.001, $\eta_{p}^{2}$ = 0.255] (11.82 ± 14.7% vs. 3.37 ± 4.07%) but the group × inhibition interaction was not significant (*p* = 0.143), which indicates that differences did not increase under incompatible conditions.

##### Flexibility

Survivors were overall slower than controls [*F*(1,41) = 10.047, *p =* 0.003, $\eta_{p}^{2}$ = 0.201] (802 ± 231 ms vs. 618 ± 181 ms), but the group × flexibility interaction was not significant (*p =* 0.841), suggesting that differences in speed between groups did not increase when flexibility was required. Survivors made more errors than controls [*F*(1,41) = 27.658, *p* < 0.0001, $\eta_{p}^{2}$ = 0.403] and the group × flexibility interaction was significant [*F*(1,41) = 11.352, *p* = 0.002, $\eta_{p}^{2}$ = 0.217], which indicates that differences in accuracy between groups increased when flexibility was required (**Figure 4B**).

#### Survivors’ Performances Compared With the Norm

Using *z*-scores, the results of the control group fell perfectly within the normal range with mean *z*-scores across tasks, varying between -0.2 and 0.3, and 0.7 < SD < 1.0. **Table 3** shows the percentage of survivors who performed within the normal range (*z* < 1), between 1 and 2 SD below (poorer than) the norm (1 ≤*z* ≤ 2), and more than 2 SD below the norm. As in the *z*-score distribution, only 2.4% falls within the *z* > 2 range, it can be concluded that a relatively large number of survivors show serious neuropsychological deficits, in particular in the domains of alertness (BS), working memory (MSL), sustained attention (SAD), and cognitive flexibility (SSV), while visuomotor function (PU, TR) is relatively spared.

**Table 3:** Percentage of survivors deviating from the norm per task.

Task^∗^	*z* < 1	1 ≤*z* ≤ 2	*z* > 2
BS (speed)	21.4	28.6	50.0
(SD of speed)	0.0	50.0	50.0
MSL (speed)	42.9	14.2	42.9
(errors)	55.2	22.4	22.4
SSV (speed)	63.6	18.2	18.2
(errors)	45.5	9.0	45.5
SAD (tempo)	0.0	46.2	43.8
(fluctuation in tempo)	23.1	30.7	46.2
(errors)	66.7	8.4	24.9
PU (accuracy)	64.3	28.6	7.1
(fluctuation in accuracy)	78.6	0.0	21.4
TR (accuracy)	42.9	49.9	7.1
(fluctuation in accuracy)	92.9	0.0	7.1

*^∗^no data for VSS (norms not available) *z* < 1 → within normal range or better than the norm, 1 ≤*z* ≤ 2 → between 1 and 2 SD below (poorer than) the norm, *z* > 2 → more than 2 SD below the norm.*

#### Relation Between Task Performance and Treatment Parameters

Duration off treatment appeared to be correlated with speed (*r* = 0.63, *p* = 0.004) and fluctuation in speed (*r* = 0.63, *p* = 0.004) in task BS, tempo (*r* = 0.56, *p* = 0.024) in task SAD, speed (*r* = 0.45, *p* = 0.023) in task MSL, inhibition speed (*r* = 0.41, *p* = 0.041), and inhibition errors (*r* = 0.40, *p* = 0.046) in task SSV. Age at diagnosis was not correlated with any task performance parameter. These results indicate that longer duration off treatment was associated with slower speed and higher fluctuation in speed in task BS, slower tempo in task SAD, slower and less accurate inhibition.

## Discussion

This study demonstrated information processing deficits in our survivors with childhood brain tumor, in particular poorer alertness, sustained attention, focused attention, working memory capacity, executive visuomotor control, and cognitive flexibility. Poorer alertness was reflected in slower and more instable response times to the imperative signal (task BS). Poorer sustained attention was demonstrated by a slower tempo, higher error rate, and in particular a higher fluctuation in tempo, the latter two measures deteriorating with TOT (task SAD). Poorer working memory capacity was reflected by a disproportionate decrease in speed when memory load was increased (task MSL) and a larger decrease in performance when a more strict recall criterion was applied, i.e., when also the correct temporal sequence had to be reproduced (task VSS). Survivors of childhood brain tumor were more susceptible to distraction than controls as reflected by the disproportionate deterioration in speed of performance in the presence of distracters (task MSL), indicating a focused attention deficit. On task SSV, the survivors were slower and made more errors, but evidence for a poor inhibition, i.e., a larger decrease in speed or increase in error rate when inhibition was required, was only trend significant or not significant, respectively. Poor cognitive flexibility in survivors was reflected in a disproportionate decrease in accuracy in error rate when flexibility was required.

The survivors were less accurate and showed a higher fluctuation in accuracy (task TR and PU) than controls especially on PU indicating poorer executive motor control. The TR task is easier to perform than the PU task because drawing a circle is an increasingly automated action and many segments can be organized and executed as a single unit (Günther et al., 2005). The trajectory in the PU task is unpredictable and the required movements are always new, necessitating more controlled processing and execution of movements reflecting the higher executive function demands. Executive functions are skills needed for purposeful, controlled activity largely mediated by the frontal cortex in the brain, which is required for reasoning, problem solving as well as planning and execution.

Two of the survivors in this study received RT and nine had received RT and CNS-directed chemotherapy (CT). Both CT and RT were neurotoxic and there was evidence that suggests both CT and RT could cause early apoptosis of oligodendrocytes, which was essential for the myelinization of axons and vasculopathy leading to ischemia (Smith, 1975; Schultheiss et al., 1995; Tofilon and Fike, 2000; Kurita et al., 2001; Reddick et al., 2006; Porto et al., 2008; Schuitema et al., 2013). Both CT and RT could damage periventricular progenitor cells thus limiting neural repair (Monje et al., 2002; Fukuda et al., 2005; Dietrich et al., 2008; Han et al., 2008; Schuitema et al., 2013). These mechanisms of damages are important to understand the late cognitive effects that could occur. Lassaletta et al. (2015) reported that a significant proportion of survivors of PF tumors require long-term special education services and have reduced rates of high school graduation and employment due to neuropsychological deficits. In addition, younger age at treatment with RT and higher dosage were reported to be associated with accelerated aging of the brain and neuropsychological dysfunction (Schuitema et al., 2013).

Armstrong et al. (2010) reported similar percentages, to those in **Table 3**, of survivors of childhood CNS malignancies with impaired attentional function, processing speed (24.0–55.2%), and impaired memory (24.6–55.2%). They studied the association between region-specific RT and neuropsychological outcome, and found that poor task efficiency and memory problems were significantly associated with irradiation to the temporal region, but not other regions. Survivors of MB and PNET (20.7% of the studied population) were reported to have difficulties with task efficiency and organization which was not seen in survivors of astrocytomas (63.9%) and other CNS tumors (15.4%). In contrast, our study cohort consisted mostly of survivors of MB who received whole-brain irradiation and supplemental focal or regional irradiation of the tumor bed. Mulhern et al. (2001, 2005) in a review reported that survivors of MB had greater IQ deficits after RT at a younger age, and poorer neuropsychological function with increasing time from completion of RT. Baron Nelson et al. (2016) reported within normal range neurocognitive functioning in most survivors of childhood brain tumor after treatment with myeloablative chemotherapy-only regimen. The findings support the detrimental effects of RT on brain tissues especially on the volume of cerebral white matter. Duration off treatment was associated with poorer cognitive function in the survivors group. Longer duration off treatment contributed to further decline in alertness, sustained attention (tempo), working memory capacity, and inhibitory control in the survivors, while we did not find any significant association of performance with age at diagnosis. This outcome is partly in line with the results of Edelstein et al. (2011) who reported that longer time since diagnosis was associated with continued decline in working memory and younger age at diagnosis was associated with lower intelligence performance and academic achievement scores, even many years after treatment had been completed. For other predictors of tasks performance such as age at diagnosis, but also for duration off treatment, a further study would be needed to determine any association.

The present study has several limitations. The number of survivors recruited into this study was small and the sample was not homogenous, i.e., the survivors varied in diagnosis with different treatment protocols/intensity. Nevertheless, we were able to identify significant group differences, frequently accompanied by large effect sizes. With the small number of survivors the correlational analyses probably lack sufficient power. This precludes to draw strong conclusions about presence or absence of significant associations, for example between age at diagnosis and cognitive deficits.

## Conclusion

Survivors with childhood brain tumor in UKMMC have significant cognitive control deficits compared to healthy controls. These deficits will certainly cause adverse impact on the quality of life and education achievement among the survivors. The cognitive damage could increase over time especially in those who received RT. We are not sure how severe the decline will be at 10 years off treatment. The next logical step is to repeat the ANT on the same cohort of survivors at a later time point to assess whether type and severity of neurocognitive dysfunction is stable or changes over time. Development of effective but less toxic treatment protocols like targeted delivery modalities are crucial to preserve cognitive function in survivors of childhood brain tumors.

## Author Contributions

HA and SL designed and performed the study. HA, SL, and LdS analyzed the data. HA and LdS wrote the manuscript in consultation with IS. All authors contributed to manuscript revision, read, and approved the submitted version.

## Conflict of Interest Statement

The authors declare that the research was conducted in the absence of any commercial or financial relationships that could be construed as a potential conflict of interest.

## Abbreviations

ANOVA: analyses of variance
ANT: Amsterdam Neuropsychological Tasks
BS: baseline speed
CCAS: cerebellar cognitive affective syndrome
CI: cranial irradiation
CNS: central nervous system
GLM: General Linear Model
IQ: intelligence quotient
IQR: interquartile range
MB: medulloblastoma
ms: millisecond
MSL: memory search letters
Nit: number of correctly identified circles
Nitco: number of correctly identified circles in the correct order
PF: posterior fossa
PNET: primitive neuroectodermal tumor
PU: pursuit
RT: radiotherapy
SAD: sustained attention dots
SD: standard deviation
SEM: standard error of the mean
SSV: shifting attentional set – visual
TOT: time-on-task
TR: tracking
VP: ventriculoperitoneal
VSS: visuo-spatial sequencing
WS: within-subject
